# Behavioural Contagion Explains Group Cohesion in a Social Crustacean

**DOI:** 10.1371/journal.pcbi.1004290

**Published:** 2015-06-11

**Authors:** Pierre Broly, Jean-Louis Deneubourg

**Affiliations:** 1 Unité d’Ecologie Sociale, Université Libre de Bruxelles, Campus de la Plaine, Bruxelles, Belgium; 2 Laboratoire Ecologie et Biodiversité, Faculté de Gestion, Economie & Science, UCLILLE, Université Lille Nord de France, Lille, France; University of Utah, UNITED STATES

## Abstract

In gregarious species, social interactions maintain group cohesion and the associated adaptive values of group living. The understanding of mechanisms leading to group cohesion is essential for understanding the collective dynamics of groups and the spatio-temporal distribution of organisms in environment. In this view, social aggregation in terrestrial isopods represents an interesting model due to its recurrence both in the field and in the laboratory. In this study, and under a perturbation context, we experimentally tested the stability of groups of woodlice according to group size and time spent in group. Our results indicate that the response to the disturbance of groups decreases with increases in these two variables. Models neglecting social effects cannot reproduce experimental data, attesting that cohesion of aggregation in terrestrial isopods is partly governed by a social effect. In particular, models involving calmed and excited individuals and a social transition between these two behavioural states more accurately reproduced our experimental data. Therefore, we concluded that group cohesion (and collective response to stimulus) in terrestrial isopods is governed by a transitory resting state under the influence of density of conspecifics and time spent in group. Lastly, we discuss the nature of direct or indirect interactions possibly implicated.

## Author Summary

Terrestrial isopods, commonly named woodlice or pill bugs, are commonly distributed soil-dwelling arthropods, particularly important in soils as macro-decomposers of leaf litter. Many species of woodlice are synanthropic and, for this reason, are easily observable in gardens, urban parks or composts. Harmless organisms and easy to raise, the woodlice represent an excellent pedagogical model in many schools, so that children may perform on these organisms various behavioral tests such as light escape or introduction to social behaviors. Indeed, woodlice are gregarious species and exhibit long phases of aggregation. Here, we propose a model based on simple rules involving calmed and excited individuals and a social transition between these two behavioural states to explain group cohesion in woodlice. This contagion model well reproduces our experimental results. Our approach provides important clues for the understanding of how social group effects and collective mechanisms may govern the stability and dispersion of aggregates in gregarious arthropods.

## Introduction

Aggregation is one of the most common social phenomena. Although it may be a response to the heterogeneities of the environment of individuals sharing the same needs, aggregation is frequently the joint response of individuals to the presence of conspecifics and the environment [1], [2]. The ultimate and proximate causes leading to animal aggregations are topics of ongoing research and are broadly known for many taxa. Response to predation risk is probably an important factor for understand groupings [3], [4] as well as feeding efficiency (e.g., [5]) or desiccation limitation (e.g., [6] for woodlice). From a mechanistic viewpoint, the use of aggregation pheromones is demonstrated in many invertebrate species [7], but many other vectors such as thigmotaxis [8] are reported.

The stability of formed groups can considerably vary both in time and space according to environmental pressure and collective or individual decision making [9], [10]. Several stimuli such as predation attacks can disturb the cohesion of aggregates and lead to more or less coordinated group effects. In this context, the interactions between individuals and particularly between naive and informed (those who perceived the risk or perturbation) individuals faced with perturbation may modulate collective reaction through an information cascade within the group. In cockroaches, the individual probability of fleeing a light perturbation decreases with the number of close and immobile individuals [11]. In many species such as gregarious harvestmen [12] or whirligig beetles [13], larger groups react faster than smaller groups. In these examples, the density-dependent dispersion of groups results from signal amplification (pheromone, collision) during signal propagation among individuals.

On the other hand, in many invertebrates, individuals chronically rest in aggregation sites and are characterised by relative phases of behavioural immobility (e.g., ants [14]; beetles [15]; solitary bees [16]; butterflies and caterpillars [17], [18]; Opiliones [19]). Individual resting time may be regulated by a basic circadian rhythm [20] but also by other biotic factors as prior individual activity [21] or presence and behaviour of conspecifics [20]. In *Drosophila*, social context increases the total amount of sleep in individuals and its effectiveness [22]. In addition, despite a strong difference in their social organisation, the individual probability of leaving the resting phase decreases with increasing numbers of individuals in the group both in the cockroach *Blattella germanica* [23] and in the ant *Lasius niger* [24].

Thus, the departure from a group can have several origins, involving external stimuli to the group or a spontaneous departure, involving social factors or not, and may act in an antagonistic manner. Group size is certainly one of the most important variables in understanding the collective mechanisms of group maintenance and how social facilitation may act [25–30]. However, essential for understanding the collective dynamics of groups and spatio-temporal distribution of organisms in environment, the social influence on group stability during the resting phase, and thereby on the ability to change state when faced with a stimulus, is poorly known and seldom discussed in the literature.

Gregarious woodlice (Crustacea: Isopoda: Oniscidea) present long aggregative phases (more often in daytime) to limit desiccation risk and short dispersal phases (often nocturnal) for solitary forage [31–35]. Aggregation in these organisms is a particularly recurrent, fast and stable process [36–40]. Aggregate initiation and formed patterns result from a dynamic trade-off between inter-attraction between conspecifics and environmental heterogeneities such as shelter [39]. In other words, aggregation in woodlice is a social phenomenon [39], [41], making it a good model for the study of gregariousness in non-eusocial arthropods (see [17]) and especially non-insect arthropods.

The collective response to a perturbation of a group composed of individuals may be a complex phenomenon to study and model. The aim of this study is a better understanding of the dynamics and mechanisms—especially social interactions—governing the group stability in gregarious species. Accordingly, we investigated group cohesion in the anthropophilic woodlouse *Porcellio scaber*, one of the most common terrestrial isopods [42]. In this context, we hypothesize that group cohesion and group dispersion may be governed by a behavioural contagion between group members. The collective response of woodlice is tested under two simple parameters: the number of individuals engaged in aggregate and the time spent in the aggregate before the perturbation. Our experimental and theoretical results show that some individuals respond faster to the perturbation and flee more quickly than others. Individuals do not interact while fleeing, but only during the aggregation period. During this period, the individuals adopt one of two behavioural states: calm or excited. Interestingly, it appears that the proportion of calm and excited individuals depends on social context and the time spent in the aggregate before the perturbation.

## Materials and Methods

### Biological material

Individuals of the common rough woodlouse *Porcellio scaber* Latreille were trapped in the gardens of the Catholic University of Lille (Northern France) and placed in culture. The culture consisted of glass boxes (410×240×225 mm) containing a humid plaster layer and soil litter (humidity≈ 80%) and were placed at 21°C under a natural photoperiod of the region.

### Experimental set-up and procedures

The experimental set-up consisted of a circular arena with a diameter of 193 mm containing at its centre a small and removable arena with a diameter of 65 mm (see S1 Fig). A sheet of white paper covered the bottom of the set-up. This sheet was changed between each experiment to eliminate potential traces of chemical deposit (see [37]). The set-up was lighted by a 40 W lamp placed 80 cm above the set-up, equivalent to a brightness of 156 lux. The small central arena (Ø 65 mm) represented a retention area in which woodlice were first introduced and kept enclosed (see the experimental conditions below), before release into the large arena (Ø 193 mm). The removal of the inner retention arena has been performed by hand in a quick movement that is perpendicular to the support of the set-up (S1 Fig). Each experiment was filmed with a Sony camera CCD FireWire—DMK 31BF03 from the release of individuals until the last individual left the retention area.

Three experimental conditions were performed:

- Groups of 10 (n = 20), 40 (n = 33), 80 (n = 21) or 120 woodlice (n = 19) were first introduced in the retention arena and kept enclosed for 300 s. After this retention time, the woodlice were released into the large arena by removing the retention arena. The departure of woodlice from retention area was then followed.
- Groups of 40 woodlice were first introduced into the retention arena and kept enclosed for 30 s (n = 15), 60 s (n = 15), 120 s (n = 15), or 600 s (n = 15). The groups of 40 woodlice kept for 300 s (see (i)) were also used. After these retention times, the woodlice were released as in (*i*), and the departure process of individuals was followed.
- Isolated individuals were successively introduced into the retention arena and kept enclosed during 30 s (n = 40), 300 s (n = 40) or 600 s (n = 40). After these retention times, the woodlouse was released as in (*i*) and (*ii*), and its departure process was followed.

### Measures and statistical analysis

Each experiment can be divided in three steps: retention, perturbation and departure.

#### 1. Retention and perturbation

To characterize the spatial patterns that were formed by individuals in the retention arena, we used several indices.

First, to estimate the number of individuals that were directly perturbed (assuming that only individuals in contact with the edge of the retention arena are disrupted during the release), the distance of individuals from the edge of the arena was measured with the Cartesian coordinates of individuals and the centre of the retention arena using the software Regressi and its plug-in Regavi (Micrelec, France).

Moreover, the group compaction was recorded just at the releasing of individuals (i.e., at t = 0 s). For this, the surface area occupied by the group in the retention area was calculated from pictures by counting the number of pixels (then converted in cm²) with Photoshop 7.0.1 (Adobe Systems Software, San Jose, California).

Finally, the angular distribution of individuals just before the release (t = 0s) was recorded using the Cartesian coordinates of individuals given by the software Regressi and its plug-in Regavi (Micrelec, France).

#### 2. Release and survival analysis

During analysis, the release of the woodlice represents t = 0 s.

Tracking of the departure process was made by counting the number of individuals present in the ex-retention area every second, thereby giving dispersion dynamics of the group. In experiments with isolated individuals, the tracking method provides individual departure time.

To quantify the departure rate, survival curves were fitted. The general decay equation is

$$\frac{dP}{dt}=-M(P)P$$ (1)

The population *P* decreases at a rate proportional to its current value and to the departure rate per individual (or probability) *M(P)*.

The master equation is the stochastic version of Eq (1). A schematic illustration of the different states of the system is given in S4A Fig. It describes the time evolution of the probability of the system to occupy each one of the discrete sets of states Ψ(P) (*P* = *P*_*0*_,…., 1, 0). *P*_*0*_ is the size of the tested population and at t = 0 s, *P* = *P*_*0*_ and Ψ(P_0_) = 1, Ψ(P_0_-1) = …,Ψ(0) = 0.

$$\frac{d\Psi (P_{0})}{dt}=-M(P_{0})(P_{0})\Psi (P_{0})$$ (2,1)

$$\frac{d\Psi (P)}{dt}=M(P+1)(P+1)\Psi (P+1)-M(P)(P)\Psi (P)$$ (2,2)

$$\frac{d\Psi (0)}{dt}=M(1)\Psi (1)$$ (2,3)

If individuals do not influence each other, *M(P)* is constant (= *k*) and the mean population decreases exponentially:

$$P=P_{0}e^{-kt}$$ (3)

In this case, the fraction of aggregated individuals at time *t* is $\text{A}=\frac{P}{P_{0}}$ and is independent of the size of the tested population. The mean time of departure is equal to *1/k*, and the half-life is equal to *ln(*2*)/k*.

If the presence of conspecifics decreases an animal’s propensity to leave, *M(P)* decreases with *P*. Therefore, the rate of departure per individual increases with time, and the mean departure time increases with the size of the tested population (*P*_*0*_). In contrast, if the presence of conspecifics increases an animal’s propensity to leave, *M(P)* increases with *P*. Therefore, the rate of departure per individual decreases with time, and the mean departure time decreases with the size of the tested population (*P*_*0*_).

Eqs (1), (2) and (3) assume that individuals are identical during the departure phase. However, during the retention phase, we assume that individuals are in non-identical behavioural states: calm and excited. *Excited* characterizes the first behavioural state of individuals during the retention phase. At the introduction into the retention arena, all individuals are assumed to be excited, i.e., in locomotion and/or in an alert state (not necessarily in movement but exhibiting movements of antennas). These excited individuals are characterized by a *fast* departure rate (i.e., a high probability per time unit to leave) during the release phase. *Calm* characterizes the second behavioural state of individuals during the retention phase. This term includes immobile individuals, with postures such as antennas folded backward or body close to the ground, a pattern that is particularly observed in aggregated individuals. These calm individuals are characterised by a *slow* departure rate (i.e. a low probability per time unit to leave) during release phase.

Excited and calm individuals may shift solitarily or under the influence of conspecifics from one state to another state.

A sum of two exponentials reveals the presence of two mean times of departure (*k*_*s*_ and *k*_*f*_) associated with the two subpopulations:

$$P=S+F=P_{s0}e^{-k_{s}t}+P_{f0}e^{-k_{f}t}$$ (4,1)

$$A=F_{s}e^{-k_{s}t}+F_{f}e^{-k_{f}t}$$ (4,2)

where *S* and *F* are the number individuals of each subpopulations at time *t*, *P*_*s0*_ and *P*_*f0*_ are the initial subpopulations and *F*_*f*_ and *F*_*s*_ are the fraction of individuals of each subpopulation at time *t*.

The corresponding stochastic equation describes the time evolution of the probability Ψ(S,F) of the system to occupy each one of the discrete sets of states (see S4B Fig):

$$\frac{d\Psi (S,F)}{dt}=-k_{s}S\Psi (S,F)-k_{f}F\Psi (S,F)+k_{s}(S+1)\Psi (S+1,F)+k_{f}(F+1)\Psi (S,F+1)$$ (5,1)

Therefore, the overall probability of observing *P* (= *S*+*F*) individuals is

$$\sum_{S=0}^{P}\Psi (S,P-S)$$ (5,2)

In this case, the mean rate of departure per individual decreases with time, and the mean departure time is independent of the size of the tested population (*P*_*0*_ = *P*_*s0*_+*P*_*f0*_) if *P*_*s0*_ and *P*_*f0*_ are independent of the total size.

At last, to test a possible collective orientation during the dispersion phase, the angular distribution of individuals was recorded in the middle of the arena, at 3 cm from the ex-retention area, using Cartesian coordinates of individuals given by the software Regressi and its plug-in Regavi (Micrelec, France).

### Data analysis

Figures and regression analyses were obtained using GraphPad Prism 5.01 software (GraphPad Software Inc.). Statistical tests were performed using GraphPad InStat 3.06 (GraphPad Software Inc.).

## Results

### Spatial analysis of dispersion

First, circular statistics show that in almost 75% of experiments, individuals did not present particular orientation during the dispersion phase (S1 Table; Rayleigh test, p> 0.05). In the other cases where a particular trajectory is observed, the orientation (mean angle) is identical to the circular distribution of individuals in the retention arena at t = 0s (S1 Table; Watson-Williams test, p< 0.05). No experiment with a random initial distribution of individuals has given an orientated pattern during dispersion. There is no inter-experimental bias in the mean angle of dispersion (Rayleigh test, R = 0.1941, p = 0.29067).

### Effects of density on collective dispersion

**Fig 1:**
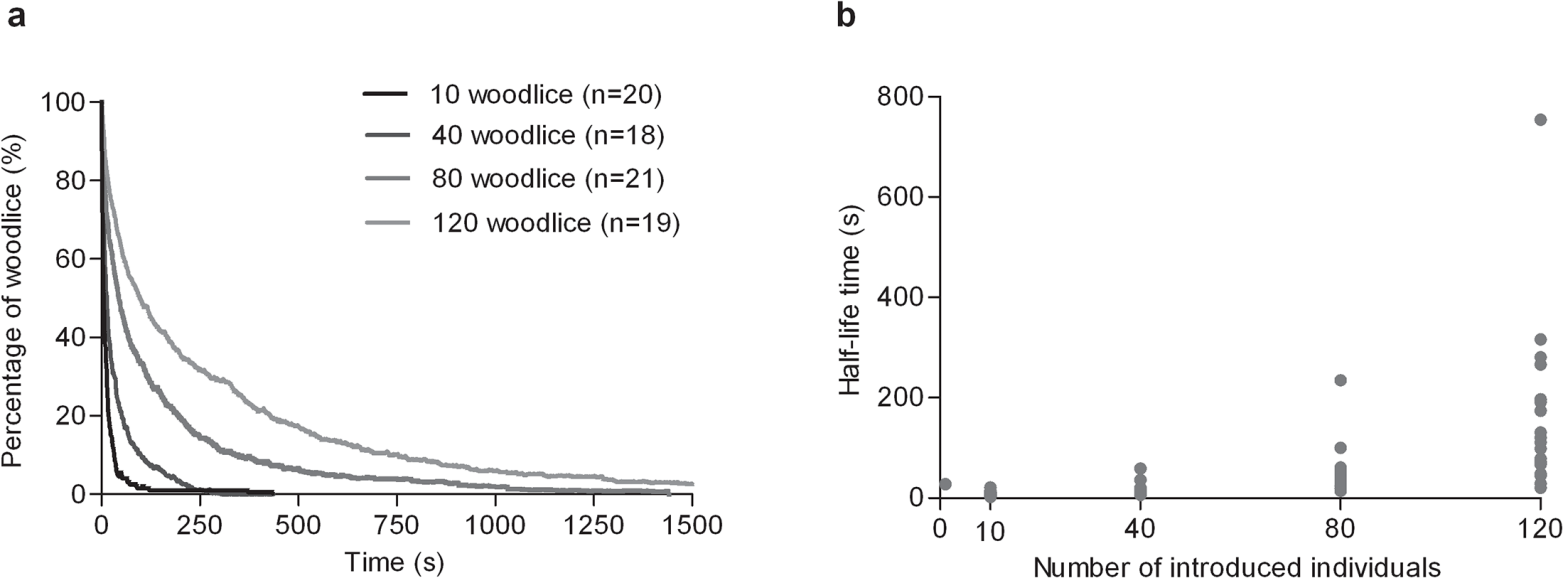
According to number of woodlice initially introduced, the dynamics of the dispersion of groups held for 300 s (a) and the time necessary to disperse 50% of the population introduced (half-life time) (b). For the experiment with 120 woodlice, only the first 1500 s were represented in Fig 1a for better visibility, but some aggregates persisted for more than 4300 s with 120 individuals.

**Fig 2:**
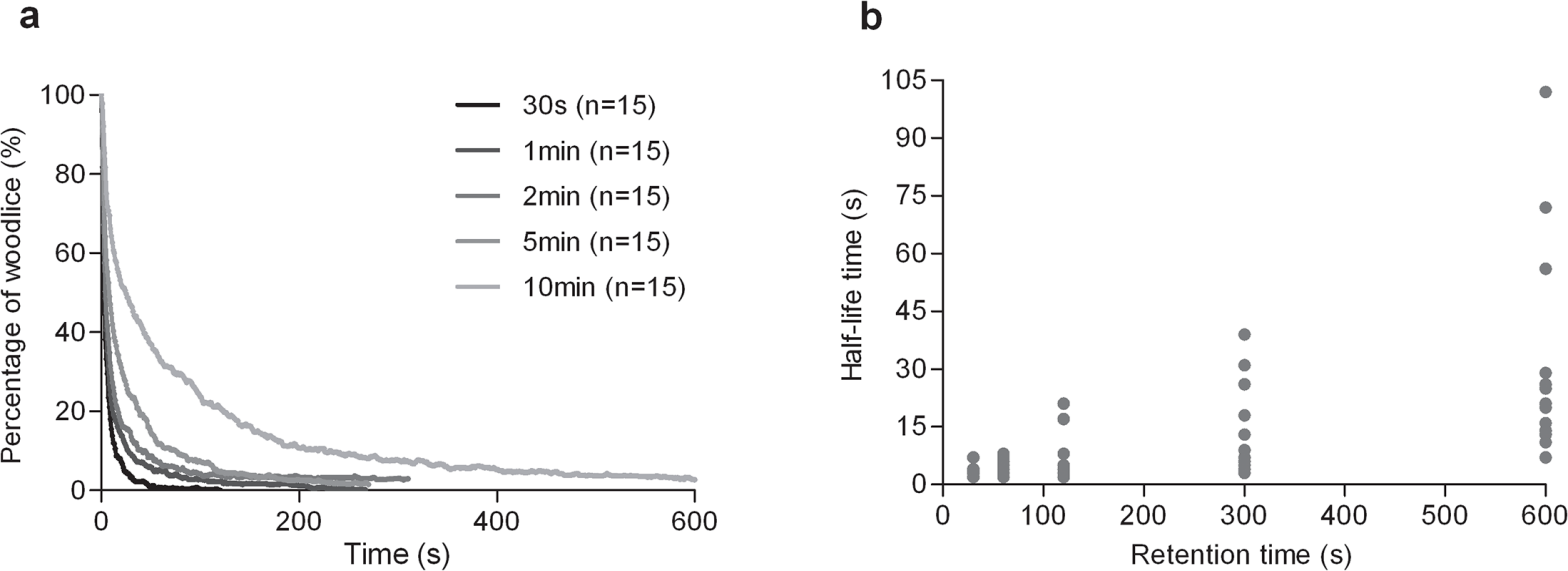
According to the initial retention time of individuals, the dynamics of dispersion of groups of 40 woodlice (a) and the time necessary to disperse 50% of the population introduced (half-life time) (b).

Whatever the experimental conditions, the dispersal dynamics from the retention area are qualitatively similar: a rapid fall in the number of individuals in the first seconds of the experiments followed by more gradual departures (Figs 1A and 2A).

However, the dynamics of dispersion are quantitatively affected by the number of individuals initially introduced (Fig 1A). The higher the number of individuals initially introduced, the slower the dynamics of dispersion (Fig 1A and 1B). The average dispersion time of the isolated individuals kept enclosed during 300 s is low (Table 1) and similar to the condition with n = 10 individuals (Fig 1B).

**Table 1:** Average departure time and half-life time (in seconds) of isolated individuals according to retention time in the central area (30, 300 or 600 s).

	30 s (n = 40)	300 s (n = 40)	600 s (n = 40)
Average departure time (s) +/- SD	10.7 +/- 20.8	27.6 +/- 56.3	31.7 +/- 72.9
Half-life time (s)	2	7	7

There is a significant difference between the half-life time of an aggregate according to the number of conspecifics introduced (Fig 1B; Kruskal-Wallis test, KW = 57.526, p< 0.0001). The half-life time of an aggregate of 10 individuals is significantly lower than those of an aggregate of 80 woodlice (Dunn test, p<0.001) and 120 woodlice (Dunn test, p<0.001). Additionally, the half-life time of an aggregate of 40 woodlice is significantly lower than with 80 woodlice (Dunn test, p<0.05) or 120 woodlice (Dunn test, p<0.001). See similar results for the ¼ life time and the ¾ life time in the supplementary material (S2A Table). In addition, the variance of the half-life time of aggregates increases with group size (Fig 1B; ANOVA test, F = 10.46, p = 0.0001). The group of 120 woodlice dispersed with a greater variability than the groups of 10 (Bonferroni test, t = 5.510, p<0.001), 40 (Bonferroni test, t = 5.069, p<0.001) or 80 woodlice (Bonferroni test, t = 3.917, p<0.01). Other conditions did not differ between treatments (Bonferroni test, p>0.05). There is a significant correlation between the coefficient of variation and the group size (Spearman test, r = 1, p = 0.0058). The coefficient of variation increases as a function of the group size according to a power law 0.49N^0.15^ (R² = 0.93).

### Effects of retention time on dispersion

In experiments with isolated individuals, increasing retention time significantly affects the departure time of individuals (Table 1; Kruskal-Wallis test, KW = 8.555, p = 0.0139). The woodlice held for 30 seconds left the retention area faster than the woodlice held for 5 min (Table 1; Dunn test, p<0.05) and for 10 min (Table 1; Dunn test, p<0.05). Note that one individual held for 600 s remained in the retention area for 2417 s. This outlier was excluded from the analysis (Grubbs test, Z = 6.05, p<0.05). The data distribution is given in the supplementary material (S2 Fig).

In addition, in group experiments, the dynamics of group dispersion are also affected by initial retention time (Fig 2A). The longer the initial retention time of individuals, the slower the dispersion after release (Fig 2A and 2B).

There is a significant difference between the half-life time of an aggregate according to the retention time of individuals (Fig 2B; Kruskal-Wallis test, KW = 40.33, p< 0.0001). The half-life time of an aggregate held for 30 s was significantly lower than those of aggregates held for 300 s (Dunn test, p<0.01) or 600 s (Dunn test, p<0.001). Additionally, the half-life time of aggregates held for 60 s or 120 s is significantly lower than in the case of aggregates held for 600 s (Dunn test, p<0.001). Other conditions did not differ from each other (Dunn test, p>0.05). See similar results for the ¼ life time and the ¾ life time in the supplementary material (S2B Table). In addition, the variance of the half-life time of aggregates increases with the retention time of individuals (Fig 2B; ANOVA test, F = 10.37, p< 0.0001). Groups held for 600 s disperse with a greater variability than groups held for 30 s (Bonferroni test, t = 5.495, p = 0.001), 60 s (Bonferroni test, t = 5.260, p = 0.001), 120 s (Bonferroni test, t = 4.940, p = 0.001) and 300 s (Bonferroni test, t = 3.650, p = 0.01). Other conditions did not differ from each other (Bonferroni test, p>0.05). There is a significant correlation between the coefficient of variation and the retention time (Spearman test, r = 0.9, p = 0.0417). The coefficient of variation increases as a function of the retention time according to 0.21t^0.25^ (R² = 0.87).

### Theoretical models

To explain our results of dispersion, several assumptions can be made on whether a social effect was or not involved in the conformation of the groups during the retention time, the releasing of individuals (i.e., perturbation) and during the departure of the individuals.

Hypotheses involving social effects during departure, different behavioural responses to the perturbation or a probability to become tired dependent on population size and retention time are not consistent with our experimental results. For this reason, they are detailed respectively in S1 Text, S2 Text and S3 Text. In brief, the retention effect of individuals during departure (i.e., an increasing departure rate) is not supported by the decreasing departure rate calculated from our experimental results (see S1 Text). In addition, the perturbation hypothesis predicting that the dynamic of dispersion is the by-product of a disturbance event and spatial conformation of individuals in the retention arena was not supported by the data of spatio-temporal distribution of individuals in the set-up (see S2 Text). Finally, the fatigue hypothesis of a fraction of individuals in the retention area, increasing with the retention time and the number of individuals, cannot reproduce the experimental results quantitatively or qualitatively (see S3 Text).

### “The Tortoise and the Hare”

First, we assume that individuals, before and during the perturbation, are in a slow or fast behavioural state with varying proportions depending on the experimental conditions. We add all the experiences assuming that the probability of departure for the slow and fast individuals are independent of the situation and that only the proportions changes with the experimental conditions.

The average survival curve of all experiments is well fitted by the two phase exponential (Eq (4), Mat and Meth), where *F*_*s*_ represents the fraction of slow individuals, *F*_*f*_ represents the fraction of fast individuals (i.e., *F*_*f*_ = 1-*F*_*s*_), *k*_*s*_ represents the inverse of mean time of departure of slow individuals, *k*_*f*_ represents the inverse of mean time of departure of fast individuals and *t* represents the time (s). *F*_*s*_ = 0.3481 (95% CI: 0.3457 to 0.3505); *F*_*f*_ = 0.6519; *k*_*s*_ = 0.003404 (95% CI: 0.003376 to 0.003431); *k*_*f*_ = 0.0687 (95% CI: 0.06772 to 0.06968); df = 4381; R² = 0.9903.

**Fig 3:**
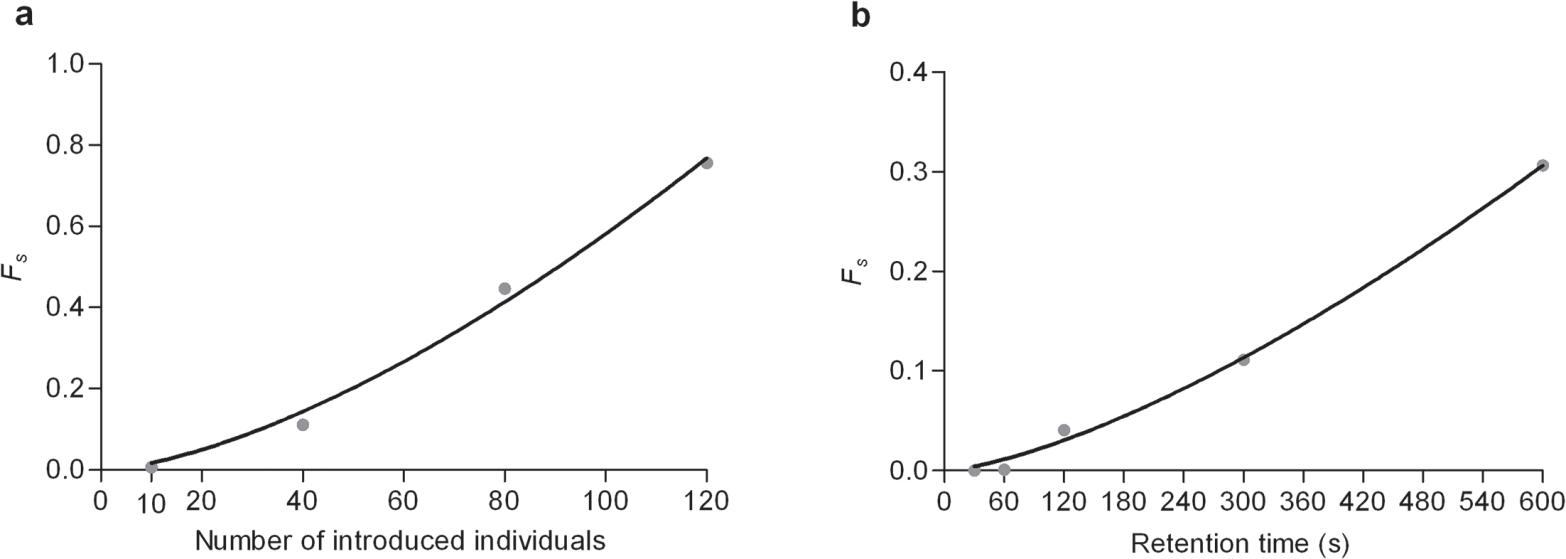
In experimental dispersions, calculated fraction of slow individuals *F*_*s*_ according to the number of initially introduced individuals (a) and retention time of groups (b).

With the constants *k*_*s*_ and *k*_*f*_ obtained previously, we calculated the mean fraction of slow individuals *F*_*s*_ and the mean fraction of fast individuals *F*_*f*_ using Eq (4) for each conditions of Figs 1A and 2A. The variation of *F*_*s*_ (and by extension *F*_*f*_) according to the number of initially introduced (*P*_*0*_) and the retention time of groups (*R*) are given in Fig 3A and 3B, respectively.

The fraction of slow individuals (*F*_*s*_) increases with group size (*P*_*0*_) according to a power law $F_{s}=5.22\;10^{-4}P_{0}^{1.5}$ (R² = 0.9930). The values given here are valid for P_0_< 120. Additionally, the fraction of slow individuals (*F*_*s*_) increases with the retention time of groups (*R*) according to a similar power law *F*_*s*_ = 3.12 10^−5^ *R* ^1.4^ (R² = 0.9964). The values given here are valid for t< 600 s.

The experimental distribution of *F*_*s*_ (see S5 Fig) was obtained by fitting each experiment of each condition one by one with Eq (4) and the constants *k*_*s*_ and *k*_*f*_ obtained previously.

During the retention phase, woodlice reduced interindividual distances in both experiments increasing number of individuals introduced (S2 Text) and retention time (S3 Fig). Based on previous studies showing the role of the interactions between conspecifics in cluster formation and mimetic behaviour [43], [44], we hypothesise that increasing spatial tightening between individuals promotes social interactions and therefore behavioural changes.

To test this hypothesis, we developed and analysed a 2-state behavioural contagion model. We assume that during the retention phase, individuals can be in two states: excited and calm; these two states lead/correspond to the slow and fast states observed during the departure phase.

The model describes the dynamic process – during the retention period – in terms of individuals adopting or leaving the slow or fast state. A schematic illustration of the model is given in S4C Fig. Individuals in the fast (slow) state can spontaneously shift in the slow (fast) state. Moreover, the individuals affect each other: the interactions between a slow and fast animal enhance the probability for the fast one to become slow or the slow one to become fast. We assume a linear relationship between the probability of transition between the state fast –> slow (slow –> fast) and the number of slow (fast) individuals:

$$\begin{array}{l} \Phi (fast\to slow)=\alpha +\beta S\\ \Phi (slow\to fast)=\mu +\omega F \end{array}$$

where *α (μ)* is the spontaneous probability that a fast (slow) individual adopts the slow (fast) state. The coefficient *β (ω)* is the coefficient of imitation: the greater the number of slow individuals (*S*), the greater the probability of transition between the fast and slow states; the greater the number of fast individuals (*F*), the greater the probability of transition between the slow and fast states.

Without social interaction *β = ω = 0*, the fraction of individuals in a slow (or fast) state is independent of the total population.

The differential equation describing the time evolution of the mean number of S is

$$\frac{dS}{dt}=\alpha F-\mu S+\beta SF-\omega SF=\alpha F-\mu S+(\beta -\omega )SF$$ (6,1)

In this study we neglected the parameter ω, and as *F+S = N* (total number of individuals):

$$\frac{dS}{dt}=(\alpha +\beta S)(N-S)-\mu S$$ (6,2)

In this paper, we worked with the stochastic version of the model (master equation). The master equation describes the time evolution of the probability *Θ(S)* of the system to occupy each of the discrete sets of states (*S = 0*, *1*,*…*., *N*). At the beginning of the retention, all individuals are fast *S = 0*, *F = N*:

$$\frac{d\Theta (S)}{dt}=(\alpha +\beta (S-1))(F+1)\Theta (S-1)-(\alpha +\beta S)F\Theta (S)+\mu (S+1)\Theta (S+1)-\mu S\Theta (S)$$ (7,1)

The mean values of S and of the fraction of slow individuals are

$$<S>=\sum_{S=0}^{N}S\Theta (S);F_{S}=\frac{<S>}{N}=\sum_{S=0}^{N}\frac{S\Theta (S)}{N}$$ (7,2)

**Fig 4:**
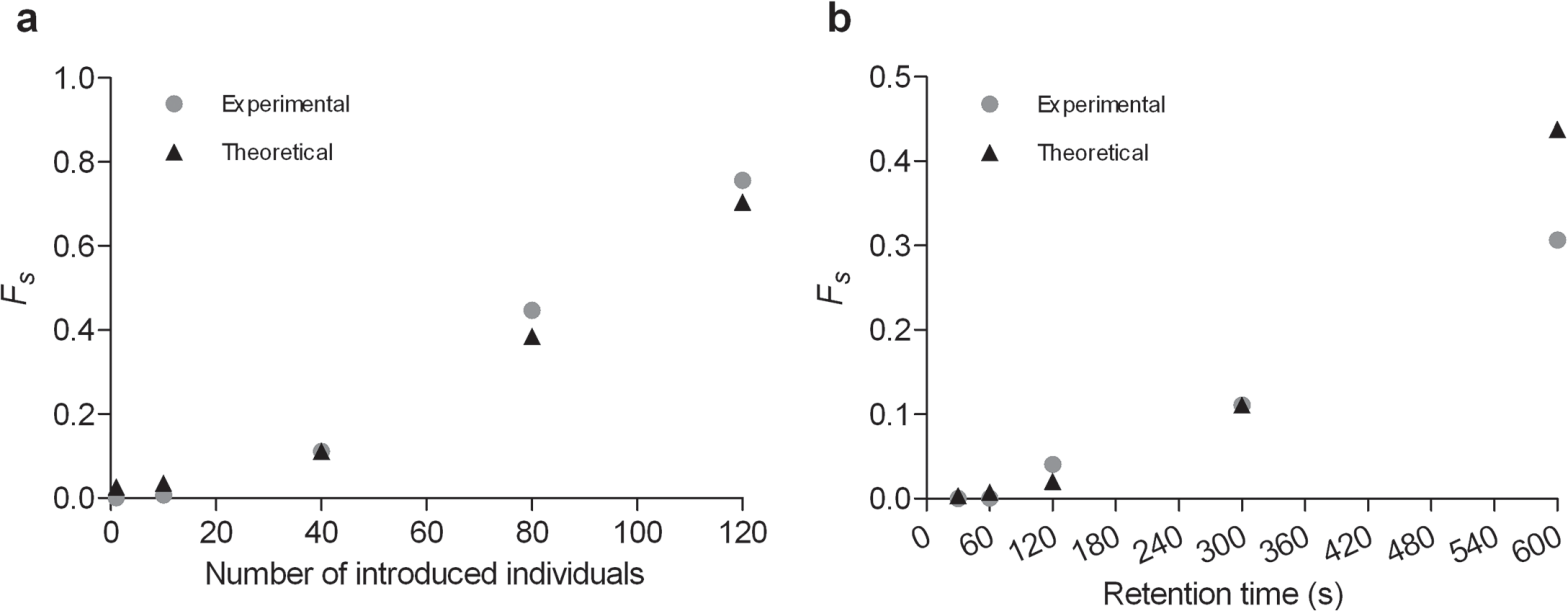
Calculated experimental and theoretical fraction of slow individuals *F*_*s*_ according to number of initially introduced individuals (a) and the retention time of groups (b).

We assumed that *α*, *β* and *μ* are constant and independent of the conditions (total number of individuals or retention time). We performed a numerical resolution of this master equation, and at the end of the retention period, the model predicts the probability *Θ (S)* of having a population of S individuals in the slow state, and a mean value of <S> or the mean theoretical fraction of slow individual (F_s_). For each condition, we searched for parameter values of *α*, *β* and *μ* for which the theoretical mean values of F_s_ is the closest to experimental mean values of F_s_. The results give *α = 10* ^*–4*^ *s* ^*-1*^; *β = 2*,*5*.*10* ^*–4*^ *s* ^*-1*^ *;* and *μ = 9*.*10* ^*–4*^ *s* ^*-1*^. For all conditions, the mean fraction of slow individuals F_s_ observed is particularly close to the F_s_ obtained with the model that is described here (Fig 4).

Moreover, for each condition, we used the Kolmogorov-Smirnov test to determine whether the distribution of experiments as a function of the slow individuals is different from the theoretical distribution *Θ(S)* for the values of the parameters (α, β, μ) giving the best fit to the mean fraction of slow individuals. The distributions are not different (S5 Fig; S3 Table, p> 0.05), except for *N* = 80 and *t* = 600 s. In the case of *N* = 80, the model overestimates the proportion of experiments with a small number of slow individuals, and in the case of t = 600 s, the model underestimates the proportion of slow individuals compared to the experimental results (S5 Fig and S3 Table). Additionally, the distribution of experiments with t = 120 s (although consistent with theoretical data; S5 Fig) does not pass the statistical test (S3 Table), but this result is only due to only one experience exhibiting a particularly long plateau phase of approximately 20 individuals (3/4 life time of 317 s when the other experiments in this condition oscillate between 3 s and 47 s).

**Fig 5:**
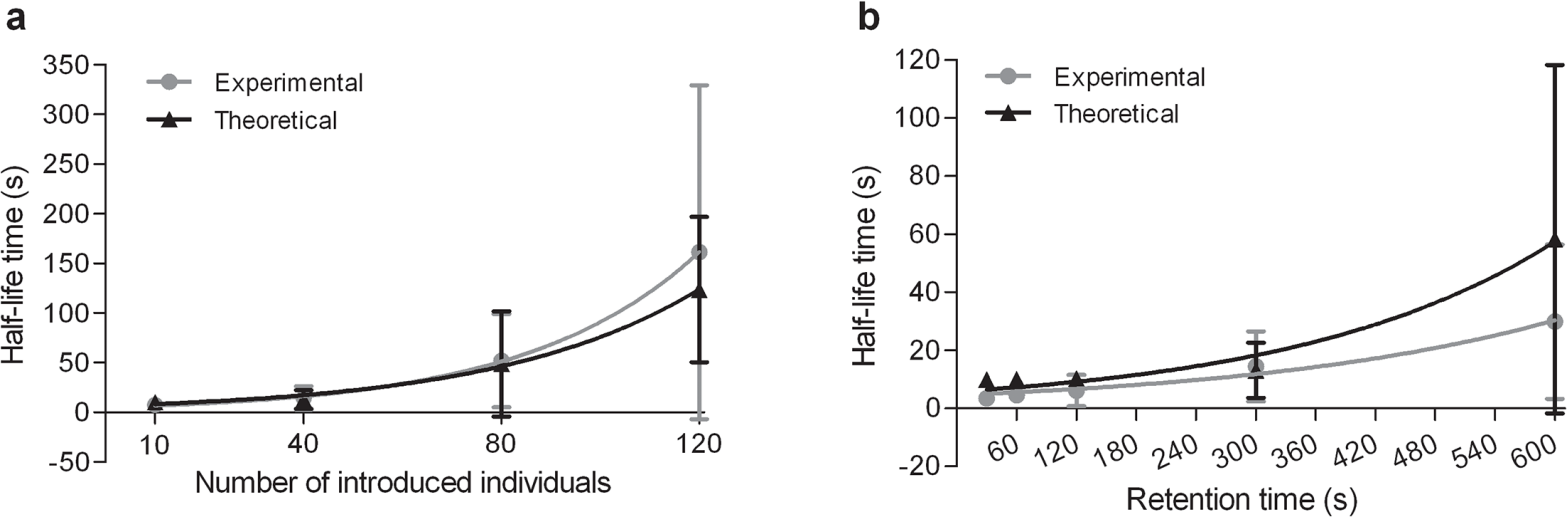
Average time necessary to disperse 50% of the population introduced (half-life time) in experiments and theoretical simulations, according to the number of woodlice initially introduced (a) and retention time (b).

**Fig 6:**
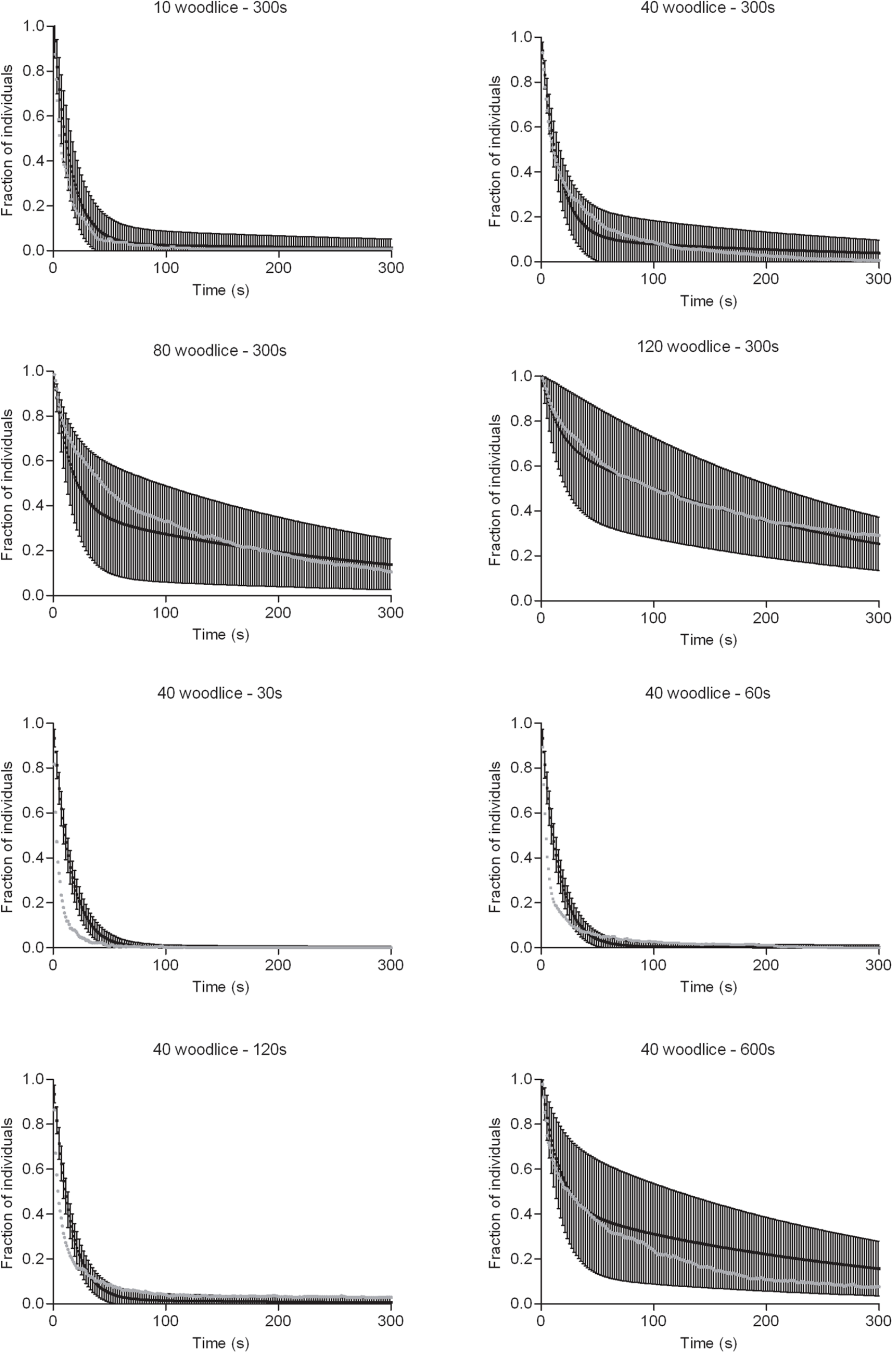
Dynamics of dispersion of experimental groups (fitting value; grey line) and simulated groups (black line) according to retention time and number of introduced woodlice.

The theoretical distribution *Θ (S)* (7,1) is also used to calculate the dynamics of dispersion (mean, variance) and mean half-life time of the dispersion (Figs 5 and 6). Eq (5,1), where *Ψ (S*, *F)* gives the probability that S and F individuals (and therefore the total population) are still in the area at time t after the release of individuals, is solved with the combinations *S = i*, *F = N- i; i = 1*, *…*, *N* at the end of the retention phase. The dispersion dynamics for each initial condition weighted by *Θ (S)* gives the theoretical distribution of the experiments according to the total population that is still in the area at time t. The theoretical results (mean half-life time, variance and dynamics of dispersion) are close to the experimental results regardless of the conditions (Figs 5 and 6; see also S5 Fig and S3 Table).

## Discussion

Fundamental rules leading to the initiation of aggregates or collective choices for resources (food sources, shelters) are now well known in many group-living invertebrates [1], [45]. In contrast, the collective mechanisms governing the stability and dispersion of group-living gregarious arthropods are poorly known. These mechanisms are nevertheless important for understanding how social influence may modulate the individual decision to stay in a place or react when faced with perturbation. In this study, we examine the collective response of groups of terrestrial isopods (Crustacea) faced with an environmental disturbance according to the number of individuals engaged in aggregate and the time spent by individuals in aggregates.

It is well known that woodlice present a strong thigmotaxis [36], [46], [47]. Contact stimuli might come from abiotic (e.g., wall of the arena) or biotic elements (e.g., conspecific). In the field, search contacts, either in micro-shelters and/or in aggregates, should be adaptive in decreasing desiccation risks [6], [48], [49]. Similarly, woodlice are strongly photonegative [36], [47], [50]. In the field, this behaviour should be adaptive to limit desiccation risks in avoiding sunlight. Here, the overall populations in the arena were exposed to a stressful condition due to light, and a part of population was exposed to a mechanical perturbation during release due to the loss of contact with the edge of the arena. Therefore, a relatively quick fleeing behaviour is not surprising.

However, the dynamics of group dispersion slows with increasing initial retention time and the number of individuals initially introduced in the set-up.

This slowdown during dispersion cannot be explained by a positive or negative feedback at work between individuals during this dispersion. They leave independently of each other. Contrariwise, we show the existence of two behavioural states of individuals or two behavioural populations: calm (slow individuals during departure) and excited (fast individuals during departure). These fractions of slow or fast individuals depend on the size of the group and the duration of retention. This dichotomy between slow and fast individuals, though it may seem excessive (it is probable that there is a greater range of excitability), is a useful simplification for a minimalist fitting. The group effect is based on the modulation of the fraction of slow and fast individuals.

At the individual level, an increase in retention time slowed the departure rate of isolated individuals probably because the stress level decreases with time spent in the retention area and increases the probability that an excited individual spontaneously shifts into a calm state. A similar phenomenon could be involved at the group level but appears insufficient to explain the group size effect. Increased group size and time spent in the group increases the potential amount of interactions between individuals [51–53] and increases the probability that individuals initiate aggregation and shift to the calm state. We assume that aggregated individuals are in the calm state. The theoretical model based on these social interactions assumes that individuals in the excited state can spontaneously shift to the calm state (and conversely) during the retention phase and that the probability of switching activity depended on the number of conspecifics and their behavioural states. This model well reproduced the experimental results. In particular, the results show that the fraction of slow individuals (*Fs*) increases with the number of initially introduced individuals and the retention time of groups. This increase in the fraction of slow individuals leads to maintaining group stability and the resting state.

Moreover, the model is able to reproduce the high intra-condition variability between experiments. This high variability strongly suggests that positive feedbacks are at work between calm and excited individuals [1], [23].

Note that the existence of a social effect during the departure phase (i.e., retention mechanisms) or non-social mechanisms (i.e. mechanical disturbance effect, increasing individual fatigue during retention, *etc*.) are not exclusive to the scenario proposed here during the retention phase; however, such complementary mechanisms should be of minor importance in contrast with the social transition between excited and calm individuals during the retention phase.

In this way, this study proposes a new understanding of aggregation in arthropods in involving the amount of time spent in the group, an often underestimated parameter in collective decision-making in view of the highly emphasised number of individuals. For example, in cockroaches, the probability of leaving a resting site decreased with the individual time spent in the site (isolated individual experiments [54]) or with the presence of conspecifics in the site (collective experiments [23], [43]), Thus, our study argues in favour of a complementarity of these two mechanisms: the individual decision to stay in the area relies on the interplay between individual resting time and the amount of interactions with settled conspecifics and their behavioural states.

Our experimental and theoretical results strongly suggest that the aggregation process during the retention phase may promote a collective entry into a behavioural quiescence or sleep-like state (see [20], [55–57]). Entrance into a sleep-like state could explain why woodlice can be observed for several dozen minutes in apparently unfavourable places (exposed at light and without cover) when isolated individuals quickly run away in similar conditions.

Our results differ from the classical vision under which larger groups react faster to perturbation than smaller groups [3] due to social facilitation and amplification of the alarm signal (e.g., mechanical [13], [58] or chemical [12] signal). In this study, woodlice in larger groups respond proportionally slower than individuals in smaller groups. This observation could highlight the particular importance of aggregation in woodlice for maintaining collective resting phases and the associated benefits (e.g., for reducing water loss [6]), in contrast with an anti-predation strategy as in many models. Woodlice, due to their cryptic way of life [59], should rarely be faced with hungry predators consuming many individuals in a short time scale in micro-habitats. Furthermore, mechanisms leading to maintaining cohesive groups in resting to reduce chronic water loss should be more adaptive than mechanisms leading to quick dispersion during rare predatory events.

However, the nature of direct or indirect interactions between woodlice in groups remains poorly understood and deserves further study. The suggested aggregation pheromone in woodlice faeces [37] probably cannot explain the rapidity of the grouping but could be involved in its stability and the transition from an excited to a calm state. The humidity generated by transpiration of a group of woodlice (see [60]) could also indirectly be involved in the collective resting process. Lastly, direct contacts between conspecifics could also be involved in these strongly thigmotactic animals [46].

In many cases, aggregation is the interplay between individual responses to environmental stimuli and to groups [1], [61–64], including in woodlice [39]. In the field, the factors inducing dispersion in woodlice are fairly well known. Woodlice in temperate environments present a (more or less endogenous) circadian rhythm of activity [32–35], [65], [66]. Foraging appears as the main activity during their short nocturnal dispersal phases [33], [34]. Thus, the combined influence of environmental factors (such as the alternation of day/night) and individual conditions (such as physiology, satiety state) probably disturbs the self-amplification process of group retention and allows for group explosion. For example, the dispersal rate increases with increasing set-up brightness (unpublished data). It would be interesting to discern which steps are affected by the brightness. We know nothing about the effect of predation on group dispersion in terrestrial isopods.

In this experimental study, we forced the formation of groups on a relatively short time scale. An important next step would be to investigate freely formed aggregates and test their stability over periods of time with a true biological significance. Additionally, characterising the behavioural state of the individuals in the aggregate would give finer information about the group effect on the resting state at the individual level.

## Supporting Information

S1 Fig Experimental set-up. (PDF)

S2 Fig Departure time in isolated individuals. (PDF)

S3 Fig Spatio-temporal patterns of groups at the release. (PDF)

S4 Fig Schematic representation of the transition probabilities between the different states of the system. (PDF)

S5 Fig Distribution of F_s_ in experiments and theoretical simulations from the retention model. (PDF)

S1 Table Random dispersion. (PDF)

S2 Table Life time of aggregates. (PDF)

S3 Table Kolmogorov-Smirnov comparisons of experimental and theoretical F_s_ distribution from the retention model. (PDF)

S1 Text Departure step. (PDF)

S2 Text Perturbation step. (PDF)

S3 Text Fatigue hypothesis. (PDF)
