# Supplementary material for: Behavioural Contagion Explains Group Cohesion in a Social Crustacean
Source: PLoS Comput Biol. 2015 Jun 11;11(6):e1004290. doi: 10.1371/journal.pcbi.1004290 (PMC4465910; doi:10.1371/journal.pcbi.1004290)
Supplement: S1 Fig — (PDF) [file pcbi.1004290.s001.pdf]

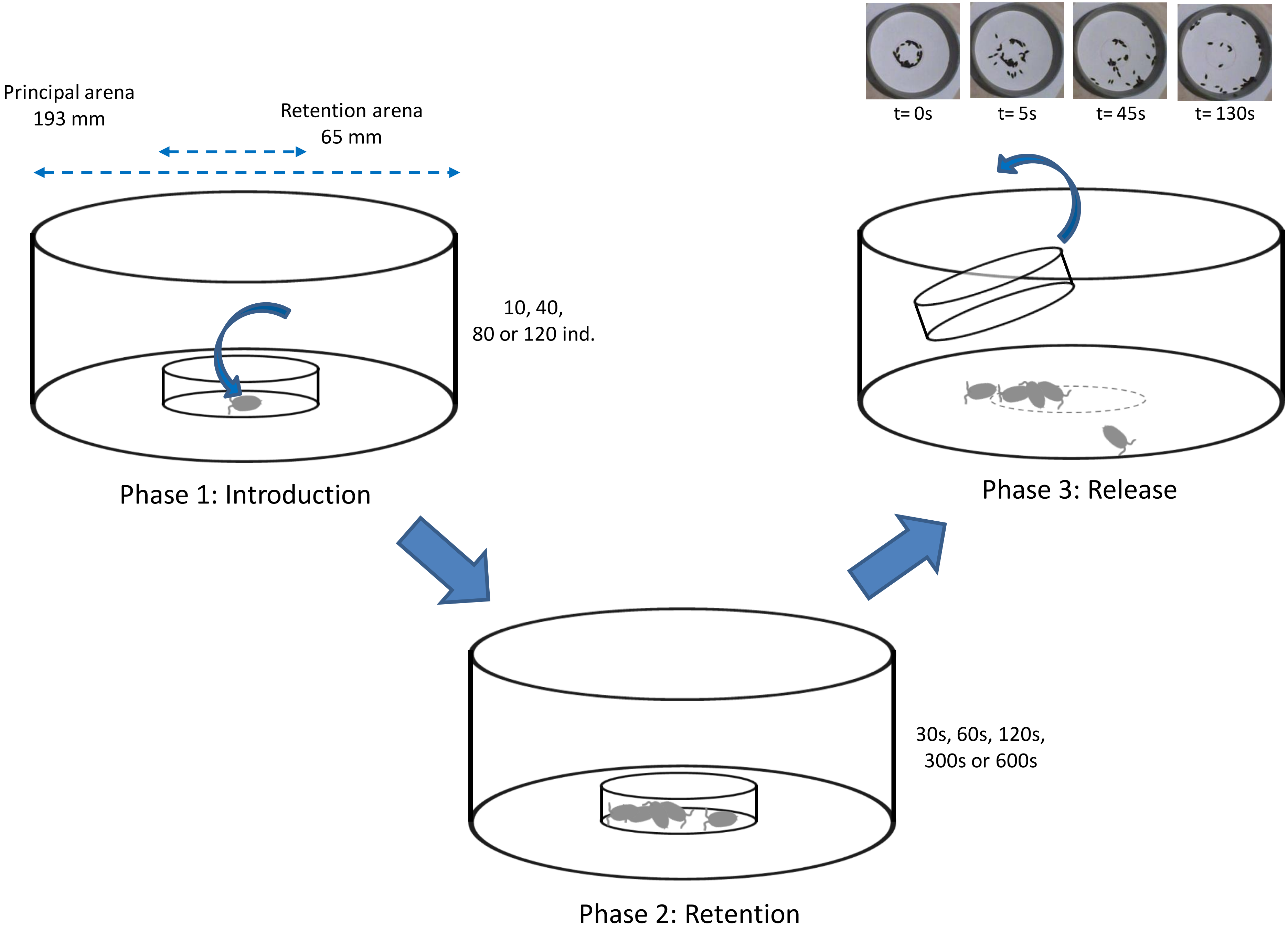
t= 0s
t= 5s
t= 45s
t= 130s
Principal arena
193 mm
Retention arena
65 mm
10, 40,
80 or 120 ind.
Phase 1: Introduction
Phase 3: Release
30s, 60s, 120s,
300s or 600s
Phase 2: Retention
