# Supplementary material for: Behavioural Contagion Explains Group Cohesion in a Social Crustacean
Source: PLoS Comput Biol. 2015 Jun 11;11(6):e1004290. doi: 10.1371/journal.pcbi.1004290 (PMC4465910; doi:10.1371/journal.pcbi.1004290)
Supplement: S2 Fig — (PDF) [file pcbi.1004290.s002.pdf]

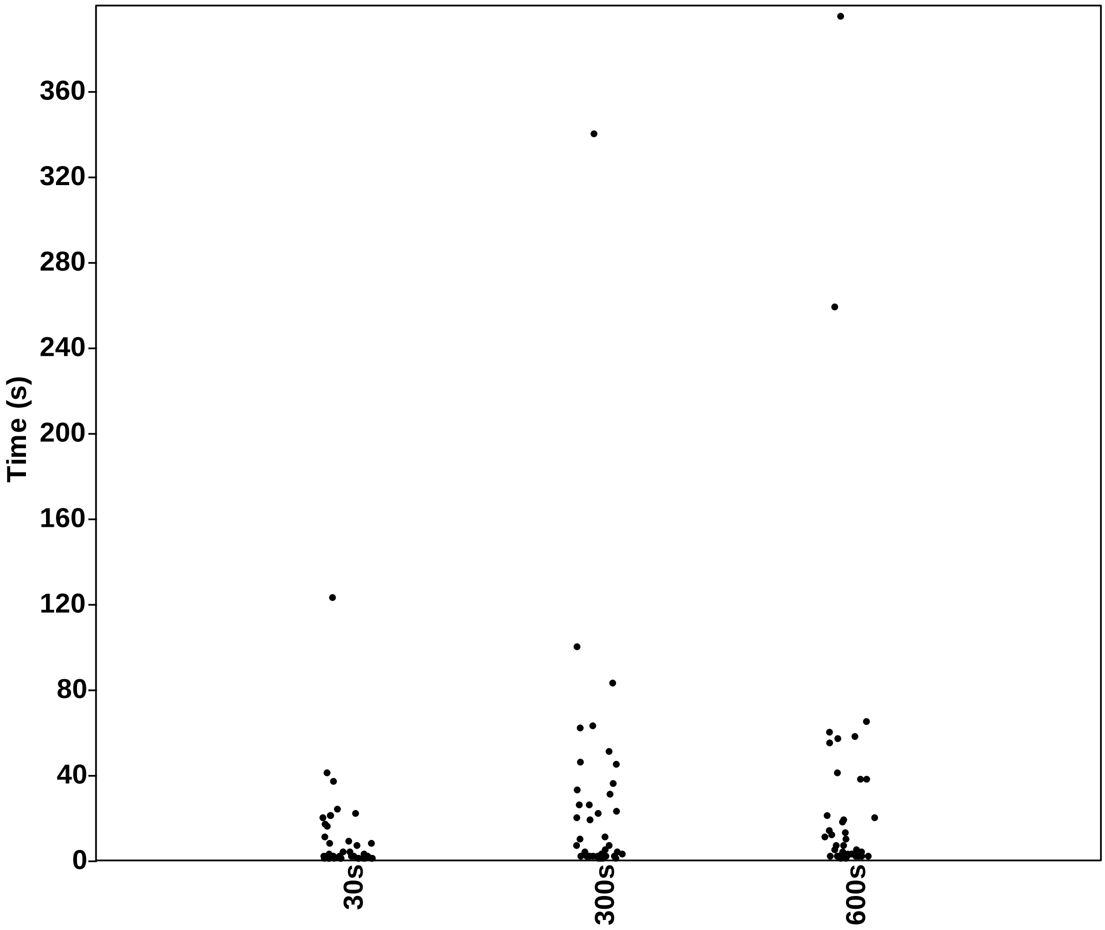

Figure S2. Jitter plot of the distribution of departure time (in seconds) in isolated individuals according to the time spent in the retention area (n = 40 for each condition).
