# Supplementary material for: Behavioural Contagion Explains Group Cohesion in a Social Crustacean
Source: PLoS Comput Biol. 2015 Jun 11;11(6):e1004290. doi: 10.1371/journal.pcbi.1004290 (PMC4465910; doi:10.1371/journal.pcbi.1004290)
Supplement: S3 Fig — (PDF) [file pcbi.1004290.s003.pdf]

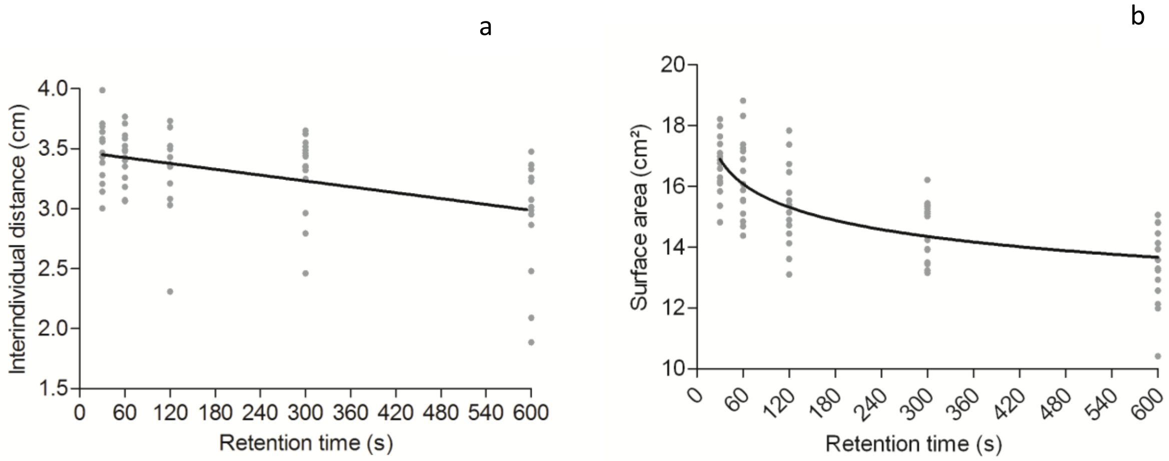

Figure S3: At the release of individuals (t=0 s), the effect of retention time on (a) interindividual distance and (b) surface area occupied by groups of 40 woodlice.

Figure S3 shows that both interindividual distance (ID in cm) and the surface area occupied by groups (*S* in cm^2) decreases with retention time (*R* in s).

The relation between interindividual distance and retention time may be fitted by the linear regression (r^2=0.2143):

$$ID = -8.10^{-4}R + 3.47$$

The slope of the regression differs significantly from 0 (F test, F=22.87, p=0.0174). There is a significant difference between average interindividual distances according to retention time (Kruskal-Wallis test follows by Dunn's test, KW= 17.238, p=0.0017). Interindividual distance in groups kept closed for 600 s is significantly lower than those of groups kept closed for 30 s (p<0.05) and 60 s minute (p<0.01).

The relation between the surface area of the group and retention time is well fitted by the nonlinear regression

$$S = S_{min} + (S_0 - S_{min})e^{bR}$$

with S_{min}= 13.02 (95% CI: 11.48 to 14.56); S_0=17.02 (95% CI: 16.28 to 17.77) b= -0.003618 (95% CI: -0.007270 to 0.00003302); df= 72; r^2= 0.5093.

There is a significant difference between average surface areas according to retention time (Kruskal-Wallis test follows by Dunn's test, KW= 39.958, p<0.0001). The average surface area of groups kept enclosed for 10 minutes is significantly lower than those of groups kept enclosed for 30 s (p< 0.001), 1 minute (p<0.001) and 2 minutes (p< 0.05). Additionally, the average surface area of groups held for 5 min statistically differs from those held for 30 s (p<0.001) and 1 min (p<0.05).

These results demonstrated that radial distance is not affected by the timing of experiments; rather, there is a stack of individuals with time (individuals gathered and overlapped), contrary to density experiments where an average significant stack appears only for more than 80 woodlice due to the physical constraint of the arena.
