# Supplementary material for: Behavioural Contagion Explains Group Cohesion in a Social Crustacean
Source: PLoS Comput Biol. 2015 Jun 11;11(6):e1004290. doi: 10.1371/journal.pcbi.1004290 (PMC4465910; doi:10.1371/journal.pcbi.1004290)
Supplement: S4 Fig — (PDF) [file pcbi.1004290.s004.pdf]

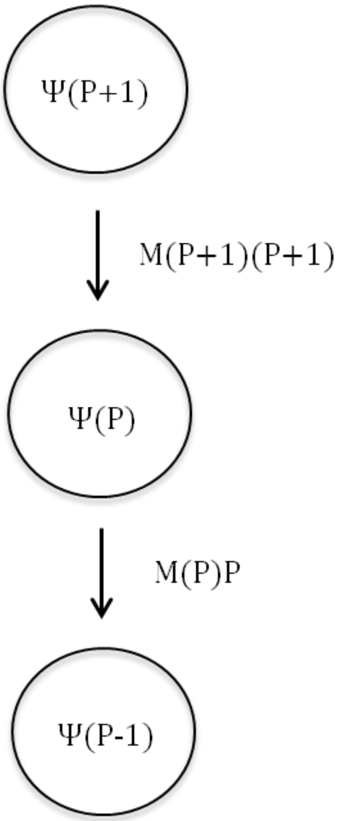

Figure S4a. Schematic representation of temporal evolution of the departure rate from equation (1).

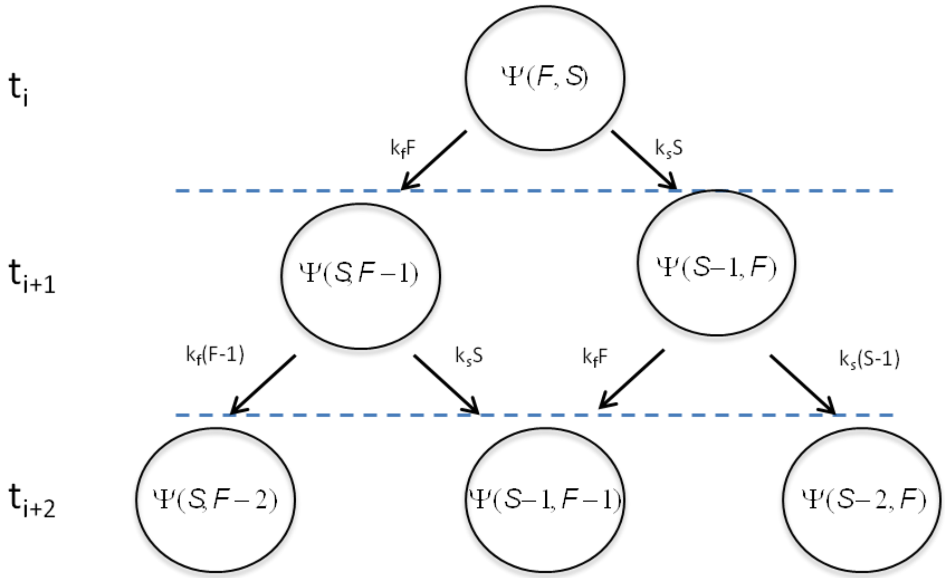

Figure S4b. Schematic representation of the departure model illustrating the transition probabilities between the different states of the system during the decay process.

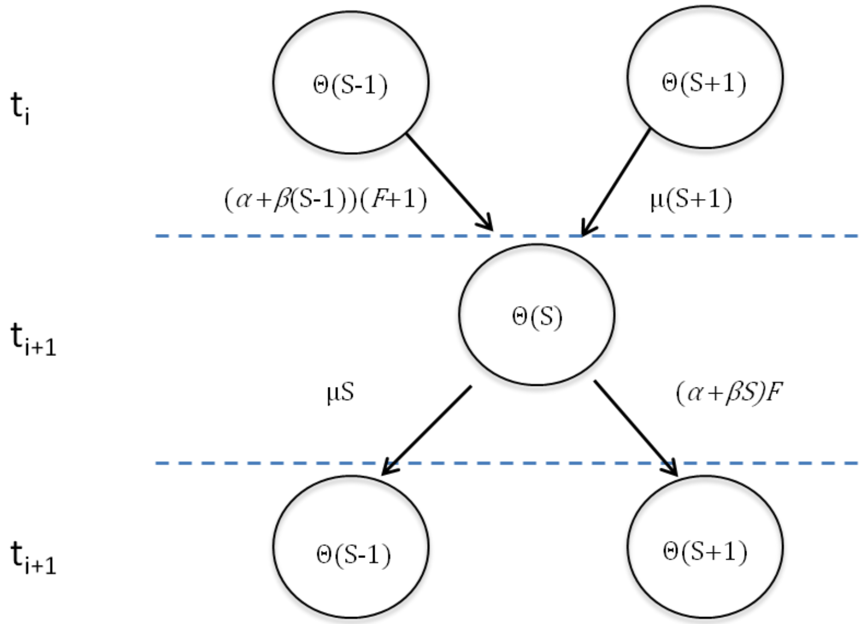

Figure S4c. Schematic representation of the retention model illustrating the transition probabilities between the different states of the system.
