# Supplementary material for: Behavioural Contagion Explains Group Cohesion in a Social Crustacean
Source: PLoS Comput Biol. 2015 Jun 11;11(6):e1004290. doi: 10.1371/journal.pcbi.1004290 (PMC4465910; doi:10.1371/journal.pcbi.1004290)
Supplement: S5 Fig — (PDF) [file pcbi.1004290.s005.pdf]

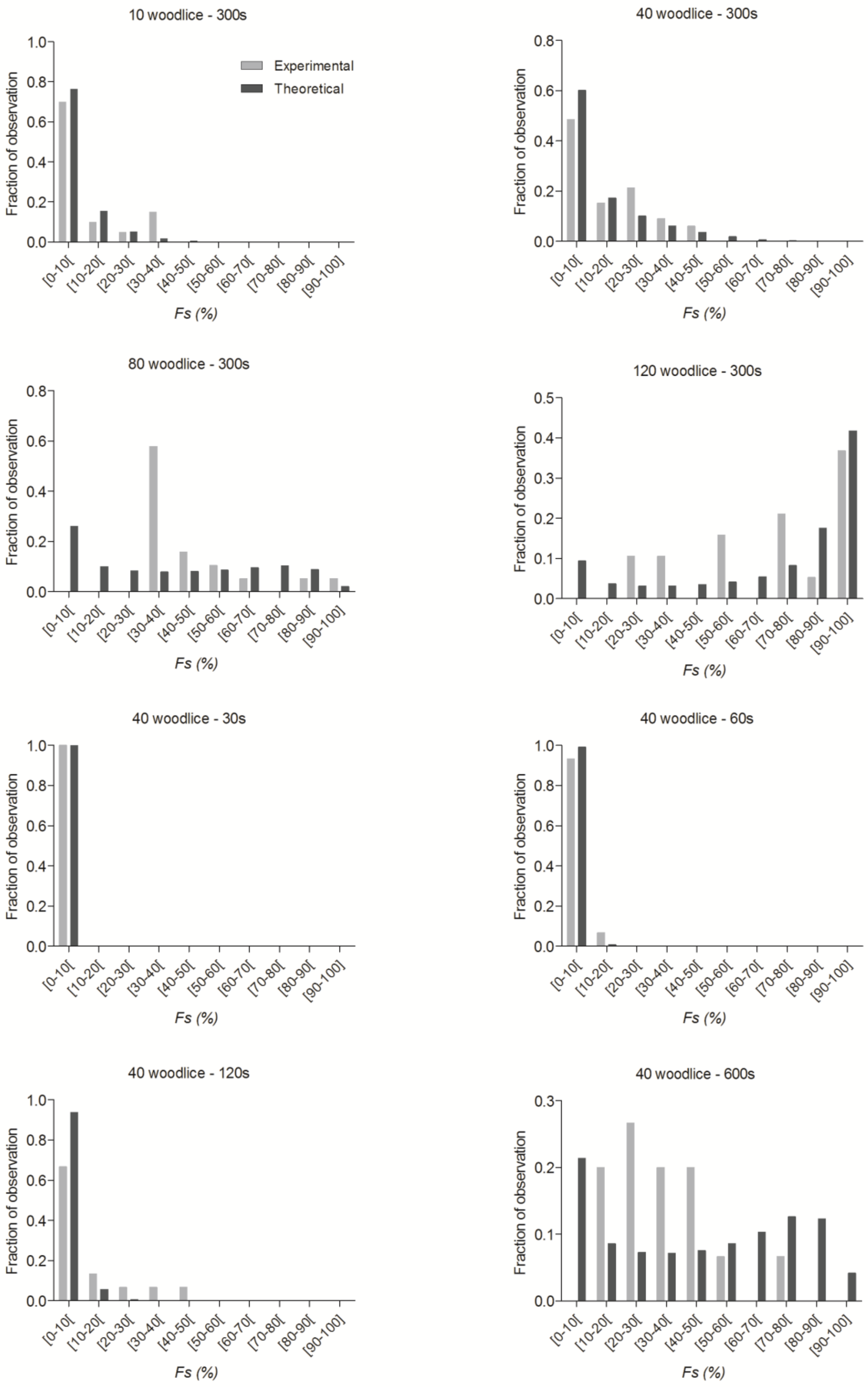

Figure S5. Distribution of the percentage of slow individuals (*Fs*) in experiments and theoretical simulations from the retention model. The experimental data were obtained by fitting each experiment individually with equation 4.
