# Supplementary material for: Behavioural Contagion Explains Group Cohesion in a Social Crustacean
Source: PLoS Comput Biol. 2015 Jun 11;11(6):e1004290. doi: 10.1371/journal.pcbi.1004290 (PMC4465910; doi:10.1371/journal.pcbi.1004290)
Supplement: S1 Table — (PDF) [file pcbi.1004290.s006.pdf]

	Orientated	Random
Number of experiments	9	24
Rayleigh statistics	$0.525 < R < 0.263$<br>$0.00001 < p < 0.037$	$0.221 < R < 0.019$<br>$0.055 < p < 0.982$

Table S1: The radial orientation of individuals during the dispersion phase of experiments included 40 woodlice that were kept enclosed for 300 s (n= 33). In this sample, 24 experiments (72%) presented a random dispersion of individuals (3 cm from the ex-retention area; Rayleigh test, $p>0.05$). The 9 other experiments presented a similar radial distribution of individuals during the dispersion phase and at t=0 s in the retention arena (Watson-Williams test, $p> 0.05$); therefore, that orientated dispersion in these experiences was mainly constrained by the initial disposition of individuals. This result suggests that there are no or few social components during the departure of individuals leading to the selection of a direction.
