# Supplementary material for: Behavioural Contagion Explains Group Cohesion in a Social Crustacean
Source: PLoS Comput Biol. 2015 Jun 11;11(6):e1004290. doi: 10.1371/journal.pcbi.1004290 (PMC4465910; doi:10.1371/journal.pcbi.1004290)
Supplement: S2 Table — (PDF) [file pcbi.1004290.s007.pdf]

	¼ life time			¾ life time		
	KW=50.217; p<0.0001			KW=56.399; p<0.0001		
	40 woodlice	80 woodlice	120 woodlice	40 woodlice	80 woodlice	120 woodlice
10 woodlice	ns	***	***	ns	***	***
40 woodlice		*	***		*	***
80 woodlice			ns			ns

Table S2a: Results of statistical tests (Kruskal-Wallis test, followed by Dunn test) comparing the ¼ life time of aggregates (i.e., time necessary to disperse ¼ of the initial population) and the ¾ life time (i.e., time necessary to disperse ¾ of the initial population) according to initial group size. *: $p<0.05$; ***: $p<0.001$; ns: $p>0.05$

	¼ life time				¾ life time			
	KW=35.637; p<0.0001				KW=44.003; p<0.0001			
	60s	120s	300s	600s	60s	120s	300s	600s
30s	ns	ns	ns	***	ns	ns	***	***
60s		ns	ns	***		ns	ns	***
120s			ns	***			ns	**
300s				ns				ns

Table S2b: Results of statistical tests (Kruskal-Wallis test, followed by Dunn test) comparing the ¼ life time of aggregates (i.e., time necessary to disperse ¼ of the initial population) and the ¾ life time (i.e., time necessary to disperse ¾ of the initial population) according to the retention time. *: $p<0.01$; ***: $p<0.001$; ns: $p>0.05$
