# Supplementary material for: Behavioural Contagion Explains Group Cohesion in a Social Crustacean
Source: PLoS Comput Biol. 2015 Jun 11;11(6):e1004290. doi: 10.1371/journal.pcbi.1004290 (PMC4465910; doi:10.1371/journal.pcbi.1004290)
Supplement: S3 Table — (PDF) [file pcbi.1004290.s008.pdf]

	10 woodlice	40 woodlice - 300s	80 woodlice	120 woodlice	30s	60s	120s	300s	600s
p	0.215	0.132	0.000	0.382	1	0.05	0.019	0.132	0.037

Table S3. Statistical results of Kolmogorov-Smirnov comparisons of experimental and theoretical *Fs* distribution for each condition. Only the distribution of experimental *Fs* in 80 and 120 woodlice cannot be considered as similar to the theoretical distribution generated from 10 000 simulations of our model.
