# Supplementary material for: Behavioural Contagion Explains Group Cohesion in a Social Crustacean
Source: PLoS Comput Biol. 2015 Jun 11;11(6):e1004290. doi: 10.1371/journal.pcbi.1004290 (PMC4465910; doi:10.1371/journal.pcbi.1004290)
Supplement: S1 Text — (PDF) [file pcbi.1004290.s009.pdf]

***Text S1.*** *Departure step*

We examined the variations of the individual departure rate with time. The results show a slower dynamic of dispersion with increasing group size (Fig. 1, Tab. S1a), suggesting an increasing retention effect of still present individuals (see Mat and Meth equation 1). In this case, the more the remaining population decreases, the more the rate of departure increases. However, the rate of departure decreases in our experimental conditions (calculated with equation 1; Fig. SI).

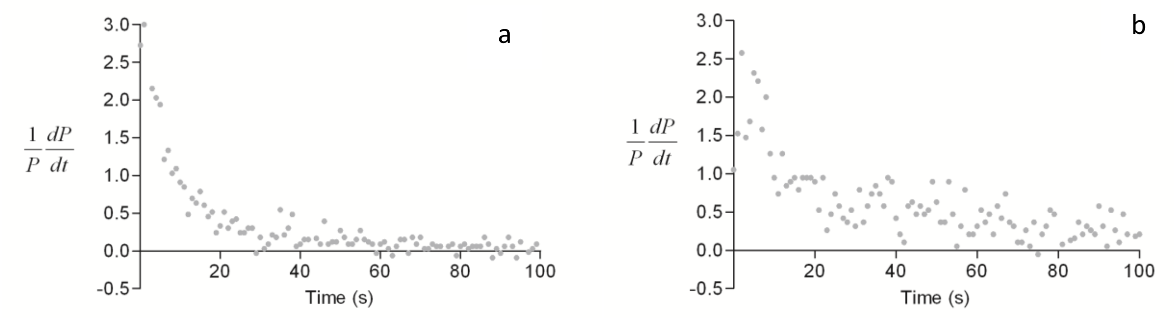


**Figure SI:** Mean departure rate as a function of the time in experiments (here with 40 (a) and 120 (b) woodlice).
