# Supplementary material for: Behavioural Contagion Explains Group Cohesion in a Social Crustacean
Source: PLoS Comput Biol. 2015 Jun 11;11(6):e1004290. doi: 10.1371/journal.pcbi.1004290 (PMC4465910; doi:10.1371/journal.pcbi.1004290)
Supplement: S2 Text — (PDF) [file pcbi.1004290.s010.pdf]

Text S2. *Perturbation step*

We explored variation in dispersion under the viewpoint of the mechanical disruption effect during release of individuals, assuming that only the individuals in contact with the edge of the retention arena are disrupted and are fast individuals while the others are slow. In other words, we hypothesise that the dynamic of dispersion is the by-product of the disturbance event and spatial conformation of individuals in the retention arena.

In density experiments, the surface area on the ground of groups increases linearly from 1 to 80 individuals and then stagnated for 120 individuals (Fig. SII). Accordingly, from 1 to 80 individuals, the stack of individuals may be negligible.

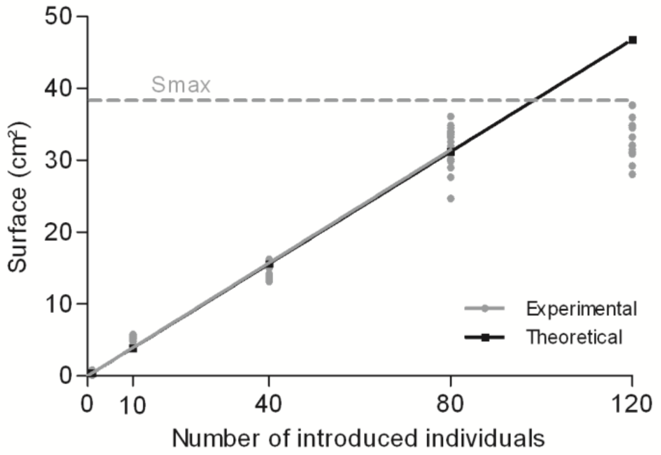

Figure SII. Relationship between the number of individuals in groups (N) and the surface area on the ground of groups (S) in experiments and in theoretical groups built under the assumption of no stacking of individuals (Surface of groups= number of individuals x individual surface). Solid lines represent the regression of the plot from 1 to 120 individuals for theoretical groups (S= 0.39N) and from 1 to 80 for the experimental group (S= 0.3933N; R^2= 0.9882). The horizontal dashed line represents the maximum surface area of a group because of the physical constraint imposed by the arena retention (38.5 cm^2).

To approximate the number of individuals in contact with the edge of the retention arena, we designed a simple geometrical model representing the spatial conformation of individuals in the arena. The perimeter of the retention arena measures 22 cm. The average length of *Porcellio scaber* is 10.4±1.6 mm (analysis previously performed on over 225 individuals). Assuming that woodlice are arranged parallel to the edge of the arena to maximise thigmotaxis and without stacking bodies (see Fig. SII, personal observations), the crown in contact with the edge of arena should contain at most 20 individuals. Hence, with these postulates, the maximal fraction of individuals possibly in contact with the edge is 1 in the experiment with ten individuals because all individuals have space in contact (10/10=1); 20/40=0.5; 20/80=0.25; (20+20)/120=0.33. For this latter condition, the surface records show stacking of 40 individuals, half of which may be in contact with the edge of retention arena.
This theoretical fraction of individuals in contact with the edge of the retention arena is compared with the fraction of fast individuals F_f obtained by the fittings previously seen ($F_f = 1 - F_s$). Figure SIIIa shows that, if the experimental fraction of fast individuals is close to the theoretical fraction of disturbed individuals on the edge of the arena for 10 and 120 individuals, the results are clearly different for 40 and 80 individuals.

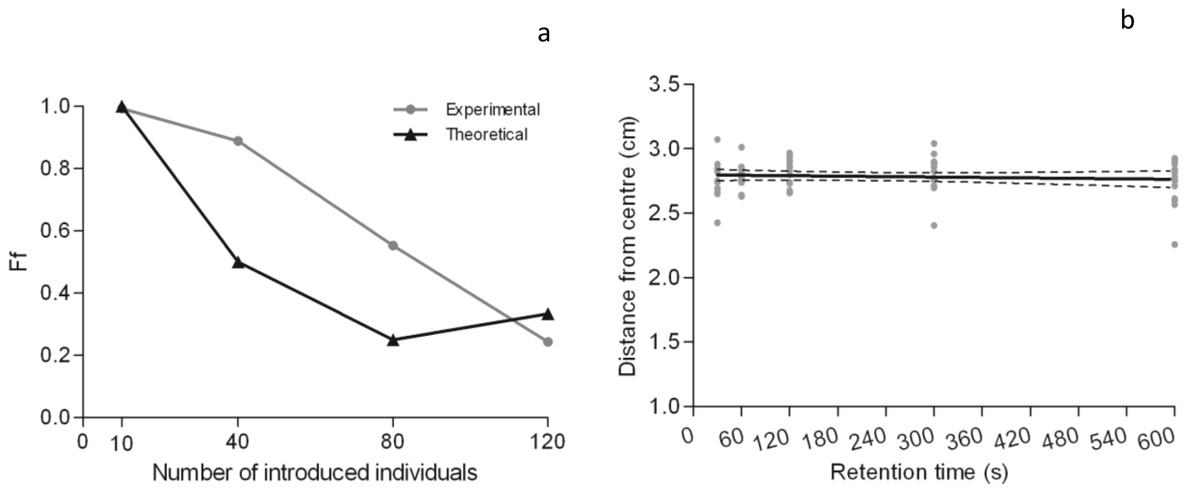

Figure SIII. (a) Experimental (fitting) and theoretical (geometrical model) fraction of excited individual F_f according to the number of introduced individuals in the retention arena. (b) Average distance of individuals from centre of the retention arena (cm) according to retention time (n=15 for each time). The solid line represents the linear regression of the scatter plot; dotted lines represent the 95% confidence interval.

In the experiments that varied the retention time (40 individuals), the small number of individuals allows the recording of the position of each individual in the retention arena. The distance of individuals from the centre of the retention arena (i.e., the closeness to the edge of the arena) does not significantly vary according to retention time (Fig. SIIIb). Indeed, the slope of the regression does not significantly deviate from zero (F test, F=0.6015, p=0.4405). In other words, the number of woodlice on the edge of retention arena which may be mechanically disrupted during the release process can be considered as constant and independent of the retention time.

Moreover, considering the width of a woodlouse to 4.8±0.9 mm (analysis previously performed on over 225 individuals), the average distance from the centre obtained (2.8 cm; Fig. SIIIb) is consistent with our geometrical model of a double ring of 2x20 individuals (in the case of 40 woodlice; see above).
