# Supplementary material for: Behavioural Contagion Explains Group Cohesion in a Social Crustacean
Source: PLoS Comput Biol. 2015 Jun 11;11(6):e1004290. doi: 10.1371/journal.pcbi.1004290 (PMC4465910; doi:10.1371/journal.pcbi.1004290)
Supplement: S3 Text — (PDF) [file pcbi.1004290.s011.pdf]

Text S3. *Fatigue hypothesis*

We tested an alternative hypothesis based on the hypothesis that individuals expend energy to escape in the retention arena. In this non-social but density-dependent scenario, we assumed that each individual has a probability per time unit (π) of becoming tired, which increases with the size of the population (N) $\pi = \alpha + \beta N$. Therefore, the number of tired individuals (Q) increases (*i*) with the time that is spent in the retention arena and (*ii*) with the size of the total population. We also assume (particularly probable under our experimental conditions with short time scales) that tired individuals cannot return to their initial condition.
With such hypotheses, the probability of an individual being in the tired state is:

$$\frac{dp}{dt} = (\alpha + \beta N)(1 - p) = \pi(1 - p)$$

at time *t= 0s, p= 0*

$$p = (1 - e^{-\pi t})$$

The probability of a group of N individuals containing Q tired individuals at time t is a binomial:

$$P(Q,N,t) = \frac{N!}{Q!(N-Q)!} p^{N-Q}(1-p)^{Q}$$

The mean number of tired individuals at time t is:

$$<Q> = N(1 - e^{\pi t})$$

and the mean fraction of tired individuals <q> (=<Q>/N) is equal to the individual probability (p).

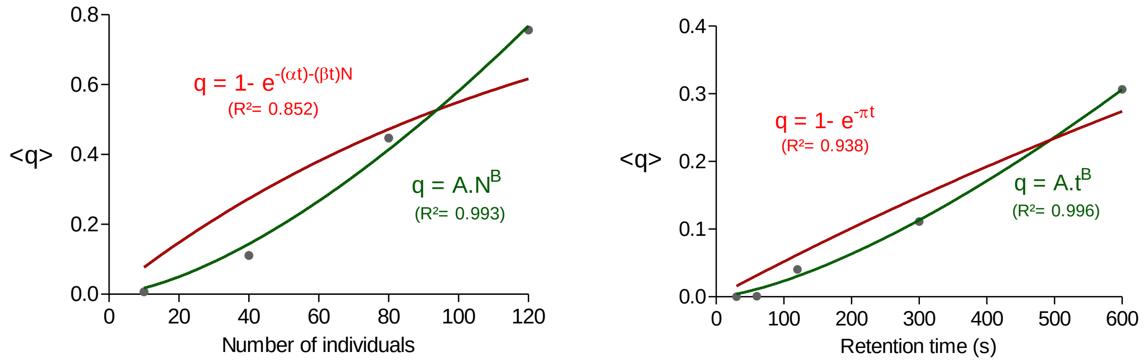

Figure SIV. Fittings of the mean fraction of tired/calm individual (q) depending of the number of introduced individuals (N) or of the retention time (t), and according to our social contagion model (green, see the Models in the results) and fatigue model described herein (red). For experiments varying the number of introduced individuals, α=0 and β= 0.0000266 for fatigue hypothesis and A= 0.000517 and B= 1.525 for social contagion model. For experiments varying the retention time, α= 0 and β=0.000013) for fatigue hypothesis and for social contagion model A= 0.0000316 and B= 1.435.

Our power relation used for modelling social contagion presents a better quantitative and qualitative fitting of experimental data than exponential relation used in fatigue hypothesis (Fig. SIV), both for experiments varying the number of individuals (F test, $p < 0.0239$, F= 40.30) or the retention time (F test, $p < 0.0060$, F= 48.97; Akaike's Information Criteria, ΔAICc= 9.360).
For each condition, we use also the Kolmogorov Smirnov test to determine whether the distribution of experiments is different from the theoretical distribution P(Q,N,t). In all cases (except for t=30 s and t=600 s), the theoretical distributions of experiments that were obtained with the fatigue hypothesis do not pass the statistical test (Fig. SV).

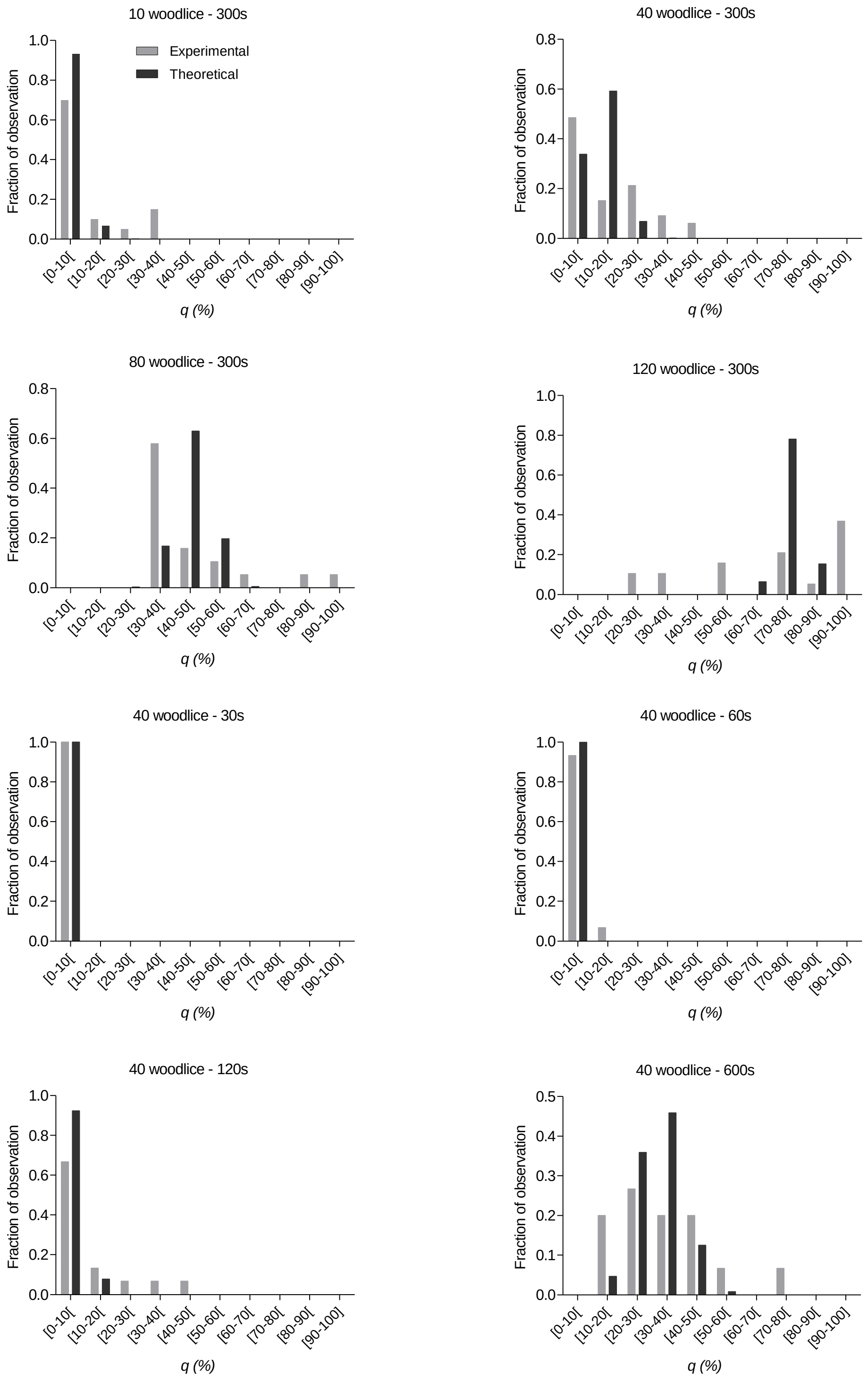

Figure SV Distribution of the percentage of tired individuals (<q> in percent) in experiments and theoretical simulations from the fatigue model. The experimental data are obtained by fitting each experiment one by one with equation 4.

In conclusion, if the fatigue hypothesis including the physiology of individuals is not completely excluded, it should be of a minor importance regarding the social mechanism that is proposed here.
